# Assessment of Self-Activation and Inhibition of Wheat Coiled-Coil Domain Containing NLR Immune Receptor Yr10_CG_

**DOI:** 10.3390/plants14020278

**Published:** 2025-01-19

**Authors:** Nan Wu, Wanqing Jiang, Zhaoxia Xiang, Raheel Asghar, Mahinur S. Akkaya

**Affiliations:** 1 School of Bioengineering, Dalian University of Technology, No. 2 Linggong Road, Dalian 116024, China; wu_nan@mail.dlut.edu.cn (N.W.); 13500518055@163.com (Z.X.); raheel@mail.dlut.edu.cn (R.A.); 2 National Key Laboratory of Agricultural Microbiology, Hubei Hongshan Laboratory, Hubei Key Laboratory of Plant Pathology, College of Plant Science and Technology, Huazhong Agricultural University, Wuhan 430070, China; jiangwanqing@webmail.hzau.edu.cn

**Keywords:** Yr10_CG_, *Nicotiana benthamiana*, cell death, plant immunity

## Abstract

Plant immunity is largely governed by nucleotide-binding leucine-rich repeat receptor (NLR). Here, we examine the molecular activation and inhibition mechanisms of the wheat CC-type NLR *Yr10_CG_*, a previously proposed candidate for the *Yr10* resistance gene. Though recent studies have identified *YrNAM* as the true *Yr10* gene, Yr10_CG_ remains an important NLR in understanding NLR-mediated immunity in wheat. In this study, we found that the overexpression of either the full-length Yr10_CG_ or its CC domain in *Nicotiana benthamiana* did not trigger cell death, suggesting a robust autoinhibitory mechanism within Yr10_CG_. However, we observed that mutations in the conserved MHD motif, specifically D502G, activated Yr10_CG_ and induced cell death. Structural modeling indicated that this mutation disrupted key interactions within the MHD motif, promoting local flexibility and activation. We further explored the effector recognition potential of Yr10_CG_ by creating chimeric proteins with Sr50 domains, revealing that both the NB-ARC and LRR domains are necessary for effector recognition, while the CC domain likely functions in downstream immune signaling. Additionally, disrupting membrane localization through an L11E mutation abolished Yr10_CG_ self-activation, suggesting a requirement for membrane association in immune activation. Our findings contribute to the understanding of CC-NLR activation and autoinhibition mechanisms, highlighting the potential of Yr10_CG_ in NLR engineering for crop resistance improvement.

## 1. Introduction

In plants, immune responses are mostly mediated by two types of receptors [1]. The first type is cell-surface-localized pattern recognition receptors (PRRs) that initiate pattern-triggered immunity (PTI) by detecting specific molecular structures in invading pathogens, called pathogen-associated molecular patterns (PAMPs), or signals from damaged host cells, termed damage-associated molecular patterns (DAMPs) [2,3]. The second type is intracellular nucleotide-binding (NB), leucine-rich repeat (LRR) receptors (NLRs). These receptors feature a variable N-terminal domain, a central nucleotide-binding and oligomerization domain (NOD), and a C-terminal LRR domain. Plant NLRs recognize race-specific pathogen effectors directly or indirectly within plant cells, initiating a process called effector-triggered immunity (ETI). This triggers a rapid programmed cell death response, termed the hypersensitive response (HR), that limits the spread of pathogen infection [4,5,6,7]. Although there have been numerous non-NLR resistance mechanisms found in crop plants, NLRs still constitute a primary class of resistance (R) genes responsible for ETI in both gymnosperms and angiosperms [8].

Plant NLRs are categorized into three main groups according to distinctive features of their N-terminal domains: CC-type NLRs (CNLs) with a coiled-coil (CC) domain, TIR-type NLRs with a Toll/interleukin-1 receptor-like (TIR) domain, and CC_R_-type NLRs with an RPW8 (resistance to powdery mildew 8)-like CC domain [9,10]. The CC domain of CNLs can also be characterized as a four-helix bundle domain and is present in various monophyletic clades of plant NLRs [11]. The CC domain of several CNLs, including Rp1-D21, Sr33, Sr50, MLA10, ZAR1, and Pvr4, can independently induce cell death when transiently expressed alone or with a tag protein in model plant leaves such as *Nicotiana benthamiana* or *Nicotiana tabacum* [12,13,14,15,16]. This suggests that the CC domain in these NLRs may act as an “executioner” or “signaling” domain upon activation. Conversely, the CC domains of other known CNLs, such as RPS5, Rx, RPM1, Pm60, and Sr35, did not trigger cell death [17,18,19,20,21].

The centrally conserved domain NOD of NLRs, also referred to as a central nucleotide-binding adaptor shared by APAF-1, R proteins, and CED-4, is NB-ARC. The NB-ARC domain functions as a molecular switch by binding and hydrolyzing nucleotides, thereby coordinating intra- and intermolecular interactions of NLR to regulate its activity. In the inactive “off” state, the NLR’s NB-ARC domain binds with ADP, and ATP binding shifts it to an “on” state, activating the NLR [11,22,23,24]. Structurally, the NB-ARC domain consists of an NB subdomain and an ARC subdomain, the latter further divided into the helical domain 1 (HD1) and winged-helix domain (WHD) [25,26,27]. Upon effector recognition, the NB domain of ZAR1 undergoes a conformational shift, releasing ADP and forming an intermediate activation state [27]. The NB-ARC domain-driven ADP/ATP exchange is crucial in the immune function of NLRs, as evidenced by functional mutants resulting from mutations in or near NB-ARC domain in various plant NLRs [19,28,29,30,31]. Conserved interactions within the NB-ARC domain stabilize ADP binding by engaging the ADP β-phosphate group with the P-loop and MHD motif [26,30,31,32]. Mutations in the highly conserved lysine residue within the P-loop motif (GxxxxGK[T/S]) disrupt ATP binding, thereby inactivating NLR signaling, as this lysine directly facilitates ADP and ATP binding in ZAR1 [26,27,33]. Conversely, the mutation of the MHD motif to MHV at the WHD subdomain end induces constitutive NLR activation in many plant NLRs, likely due to weakened ADP binding in the ARC subdomain, promoting domain reconfiguration that facilitates ATP binding and the activation of the NLR [23,28,34,35,36,37,38].

The LRR domains in NLRs are characterized by a recurring sequence pattern of leucine or other hydrophobic amino acids (LxxLxLxxNxL), which form a parallel β-sheet and an arc-shaped structure [25,26,27]. These domains serve two primary functions: the autoinhibition of NLR activity and the detection of pathogen effectors. The LRR of ZAR1 inhibits ZAR1 by sequestering the NLR in a monomeric state [26]. In *Solanum lycopersicum* (tomato), the helper coiled-coil NLR required for cell death 2 (NRC2) forms oligomers that stabilize its inactive state. The LRR domain of NRC2 binds cofactors such as inositol phosphates, which are essential in its autoinhibition. Mutations that disrupt these interactions enhance pathogen-induced cell death, demonstrating the regulatory role of the LRR domain in maintaining NRC2 in an inactive conformation [39]. Hypervariable, solvent-exposed residues in the LRR domain allow for diverse intra- and intermolecular interactions, enhancing flexibility in binding [25]. Constitutive activation in several NLRs, such as RPS5, MLA10, Pvr4, RPP1A, and L6, has been induced by the deletion or swapping of LRR domains [16,35,37,40,41]. LRR domains can recognize effectors directly or through intermediaries, known as guardees, which are targeted by effectors [26,41,42,43,44]. For example, *Arabidopsis* CC-NLR ZAR1 detects its effector indirectly through its guardee and decoy RKS1, whereas wheat CC-NLR Sr35 directly interacts with its cognate effector AvrSr35 via its LRR domain [26,45,46]. Effector binding in Sr35 disrupts its autoinhibition and initiates conformational changes that activate immunity [45,46].

Oligomerization or self-association is considered to be a common mechanism for activating NLR, playing a crucial role in the formation of functional complexes that mediate immune responses [47]. In recent studies, oligomeric NLRs such as ZAR1, Sr35, RPP1, and Roq1 have been shown to form pentameric or tetrameric complexes that are crucial to their cell death and resistance functions [27,43,44,45,46]. Sequential conformational changes, along with ADP/ATP exchange and oligomerization, are essential in NLR activation [11,31]. Effector recognition induces a steric clash in the NB domain, causing structural alterations that facilitate ADP/ATP exchange by rotating the NB outward. Following ATP binding, NLRs undergo further structural changes, including the rotation of the WHD-LRR fragment, leading to the assembly of oligomerization and the activation of immune responses [26,27]. Oligomerization may bring N-terminal signaling domains close together, allowing them to form higher-order functional structures that play roles as scaffolding, channels, or NADase holoenzymes [31]. For instance, during activation triggered by pathogen virulence factors, ZAR1 forms a higher-order oligomeric complex known as a “resistosome”. In this resistosome, the primary helix of each CC domain’s four-helix bundle forms a funnel-like structure, potentially creating pores in the plasma membrane [27]. Supporting this concept, studies have shown that activated pentamers of ZAR1 and Sr35 accumulate at lipid bilayers, acting as nonselective cation channels in vitro [11,32]. In 2014, a decade ago, a gene (AF149112, TraesCS1B03G0000200), was cloned and was believed to have Yr10 resistance activity for yellow rust [48] (also known as stripe rust, a wheat disease caused by *Puccinia striiformis* f. sp. *tritici* (*Pst*)) encoding a canonical CNL protein, which was the first wheat CNL gene cloned. Nevertheless, this gene was considered a *Yr10* gene for a long time until 2018. The studies conducted with many positive cultivars for *Yr10* found them susceptible despite having the gene; *Yr10*-cDNA-positive transgenic plants were also susceptible to the Yr10 avirulent *Pst* race, indicating that AF149112 cannot underlie *Yr10* resistance activity [49]. It was then referred to as a ‘Yr10 candidate gene’ (*Yr10_CG_*). Only recently, another gene named *YrNAM* (OP490604, TraesCS1B03G0003600 LC.1 & TraesCS1B03G0003500 LC.1), which encodes a ‘No Apical Meristem’ (NAM) domain and a Zinc Finger (ZnF)-BED domain, was shown to host Yr10 resistance, leading to claims that it was the true *Yr10* gene [49,50,51]. Although *Yr10_CG_* is not the causal gene for *Yr10*, it represents an already-cloned NLR gene in wheat, making it valuable in investigating the molecular mechanisms underlying NLR function in this crop.

**Figure 1:**
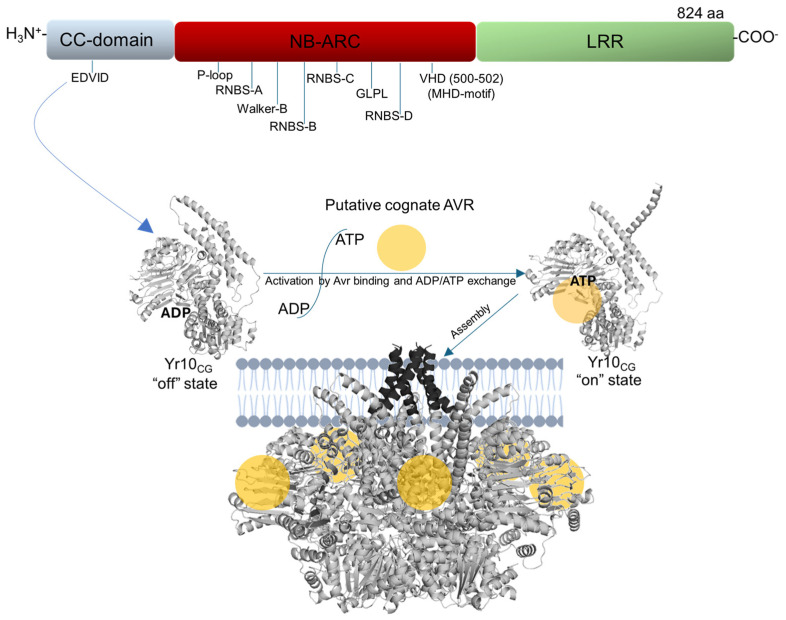
Schematic representation of Yr10_CG_ structural domains and presumed activation mechanism. Yr10_CG_, a canonical CNL protein including the CC domain, NB-ARC domain, and LRR domain. The inactive state is maintained through ADP binding in the NB-ARC domain, with the LRR domain providing autoinhibition. Upon putative Avr recognition and ADP/ATP exchange, conformational changes are triggered in the Yr10_CG_. This process leads to oligomerization, forming a pentameric complex at the plasma membrane. The CC domains assemble into a funnel-like structure, potentially creating a pore for calcium ion influx, which transduces immune signaling and leads to programmed cell death (hypersensitive response).

Our work was designed to test the impact of mutations introduced at the MHD motif and the CC domain of Yr10_CG_ in plants. MHD is a structurally critical region in keeping the CC-NLRs in inactive mode in the absence of biotic stress. However, it is constitutively expressed to switch into the active state upon infection with a pathogen secreting a cognate effector (avirulence factor (Avr)) by physical interaction. The association of Avr induces a conformational change in the NLR protein. This change is activated by ATP binding, leading to the projection of the CC-domain and the assembly of a pentameric complex (resistosome) in the cell membrane. The resistosome introduces pores in the membrane, causing calcium influx. This signal transduction triggers programmed cell death, which prevents the propagation of the pathogen and achieves resistance. Various MHD mutants of CC-NLRs cause conformational change, similarly to the recognition of Avr by CC-NLRs, which is known as “on state” conformation or “activate” state. The mutations that we conducted in the MHD motif were able to induce cell death. This information is critical, especially together with the overexpression of *Yr10_CG_* that cannot trigger cell death. Indeed, the fact that cell death can be observed in the non-host species *Nicotiana benthamiana* allows experiments to be conducted to search for cognate Avrs, since transient over-expression can be robustly conducted in *Nicotiana benthamiana*, unlike in native host, wheat. Additionally, the suppression of the cell death phenotype observed by introducing the L11E mutation into a self-activating mutant, D502G, allows further experimentations to determine downstream interactions or time and space analyses of the formation of the resistosome without hindrance due to cell death [52]. The chimeric NLR that we engineered, named *Yr10_CG_^syn1^*, which combined the CC and NB-ARC domains from Yr10_CG_ with the LRR domain from Sr50, did not induce cell death when co-expressed with AvrSr50. In contrast, the co-expression of *Yr10_CG_^syn2^*, a chimeric NLR containing the CC domain from *Yr10_CG_* and the NB-ARC and LRR domains from Sr50, with AvrSr50, resulted in cell death, similarly to the co-expression native pair Sr50/AvrSr50. These findings suggest that the NB-ARC and LRR domains are not dispensable in AvrSr50 recognition, while the CC domain can be switched to the CC domain in Yr10_CG_ (Figure 1). The critical findings of this study can guide the engineering of Yr10_CG_ for broader applications in wheat breeding.

## 2. Results

### 2.1. Yr10_CG_ Is a CC-Type NLR Immune Receptor

**Figure 2:**
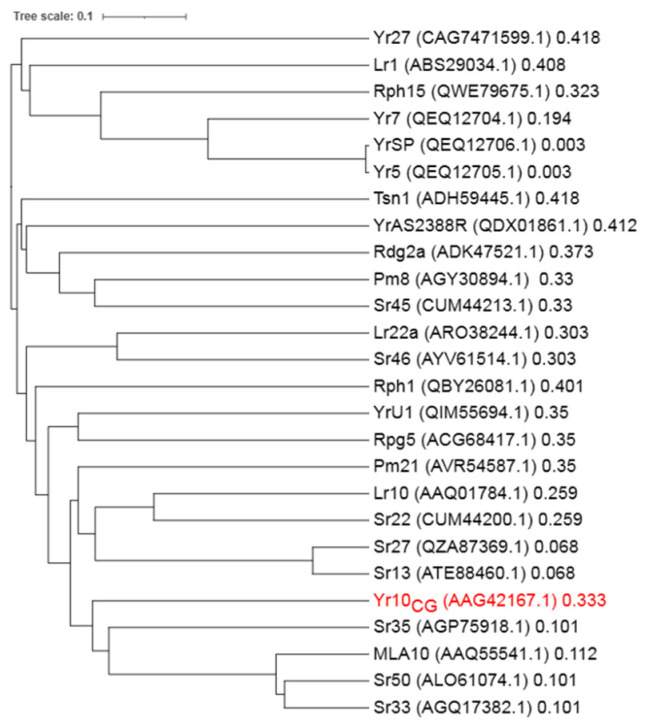
Phylogenetic tree of Yr10_CG_ and cloned NLRs from *Triticeae*. Phylogenetic tree of Yr10CG and cloned NLRs from *Triticeae*. The protein accession numbers from GenBank are indicated in parentheses. The tree was generated based on multiple sequence alignment (MSA) using the neighbor-joining method, and the distance scale is shown to represent evolutionary distances between sequences.

**Figure 3:**
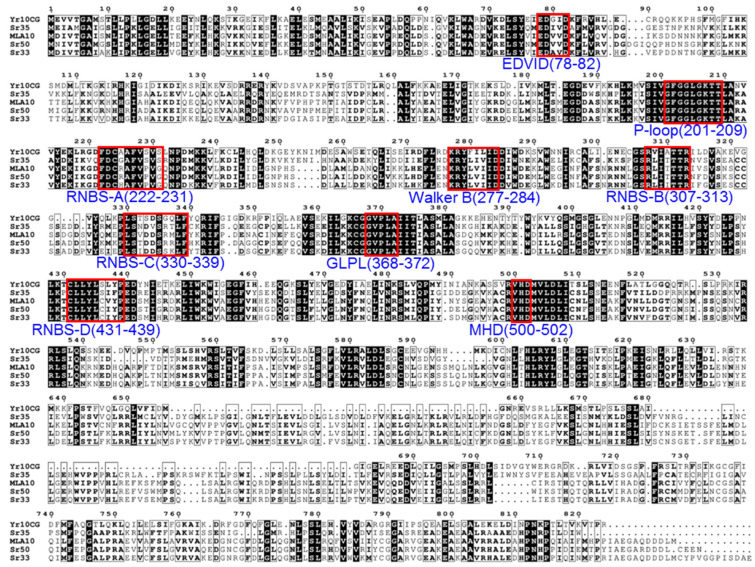
Amino acid alignment of Yr10_CG_ with Sr35, MLA10, Sr50, and Sr33. Conserved motifs are highlighted within red boxes, with motif position in Yr10_CG_ labeled below the sequences. Amino acids being on a black background indicates 100% similarity, while amino acids being in bold indicates over 70% similarity based on physico-chemical properties. The associated NLRs and their protein sequence GenBank accession numbers are as follows: Yr10_CG_ (AAG42167.1), Sr35 (AGP75918.1), MLA10 (AAQ55541.1), Sr50 (ALO61074.1), and Sr33 (AGQ17382.1).

Although Yr10_CG_ has been identified as a CNL, its specific motif features have not yet been fully characterized. By comparing the protein sequences of Yr10_CG_ with other identified NLRs in *Triticeae*, we found that Yr10_CG_ showed high similarity with Sr35, MLA10, Sr33, and Sr50 (Figure 2). Subsequently, the protein sequences of Yr10_CG_, Sr35, MLA10, Sr33, and Sr50 were aligned to identify potential motifs at specific positions (Figure 3). Among these, the P-loop, GLPL, and MHD motifs are highly conserved. The canonical MHD motif appears as VHD in Yr10_CG_, Sr35, MLA10, Sr50, and Sr33, where the methionine (M) is replaced by a valine (V). This analysis provides further insights into the motif composition of Yr10_CG_, suggesting structural or functional regions that could contribute to its immune response mechanisms.

### 2.2. Neither the Overexpression of Yr10_CG_ nor Its CC Domain Triggers Cell Death

**Figure 4:**
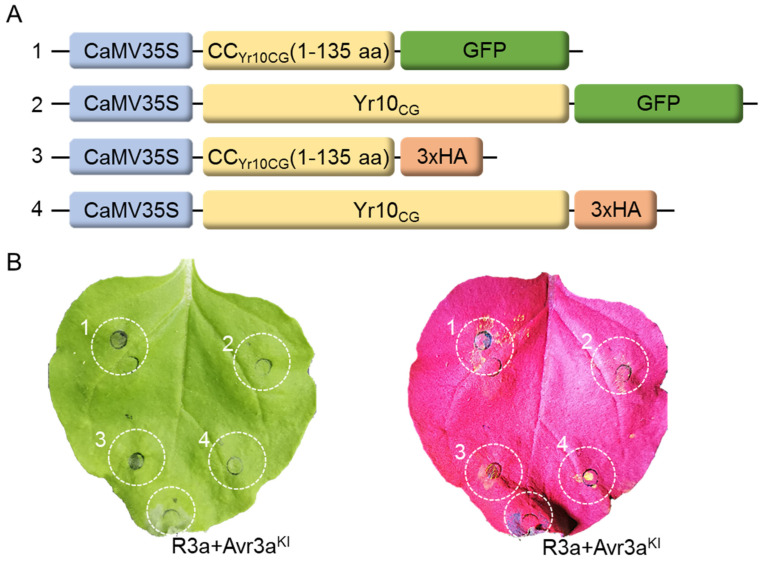
Transient expression of Yr10_CG_ and its CC domain in *Nicotiana benthamiana*. (**A**) Schematic diagram of the expression vectors for Yr10_CG_ and the CC domain of Yr10_CG_ (135 amino acids, referred to as CC_Yr10CG_), each fused to either GFP or a 3×HA tag. (**B**) *Agrobacterium* (GV3101) containing the Gateway constructs from (**A**) was infiltrated into 6-week-old *Nicotiana benthamiana* leaves. Hypersensitive responses were detected at 24 h post infiltration (hpi). The cell death was visualized under excitation at 440–460 nm using the LUYOR-3415RG Hand-Held Lamp with a 500 nm filter. Co-infiltration of R3a+Avr3a^KI^ showed cell death as a positive control.

Some studies have reported that transiently expressing either full-length NLR proteins or their CC domain in *Nicotiana benthamiana* leaves can induce cell death [14,37,53]. However, our research found that neither the transient expression of the full-length Yr10_CG_ protein nor its CC domain in *Nicotiana benthamiana* leaves triggered cell death, regardless of whether the C-terminus was fused to a larger fluorescent protein like GFP or a smaller tag protein like 3×HA (Figure 4). To ensure the reliability of this result, we included R3a+Avr3a^KI^ as a positive control, which consistently induced hypersensitive response (HR)-mediated cell death under the same experimental conditions (Figure 4). The absence of cell death in Yr10_CG_ constructs may suggest that Yr10_CG_ has a strong internal autoinhibitory mechanism, and its CC domain alone is insufficient to initiate immune signaling.

### 2.3. Autoactive Mutant Yr10_CG_-D502G Induces Cell Death

**Figure 5:**
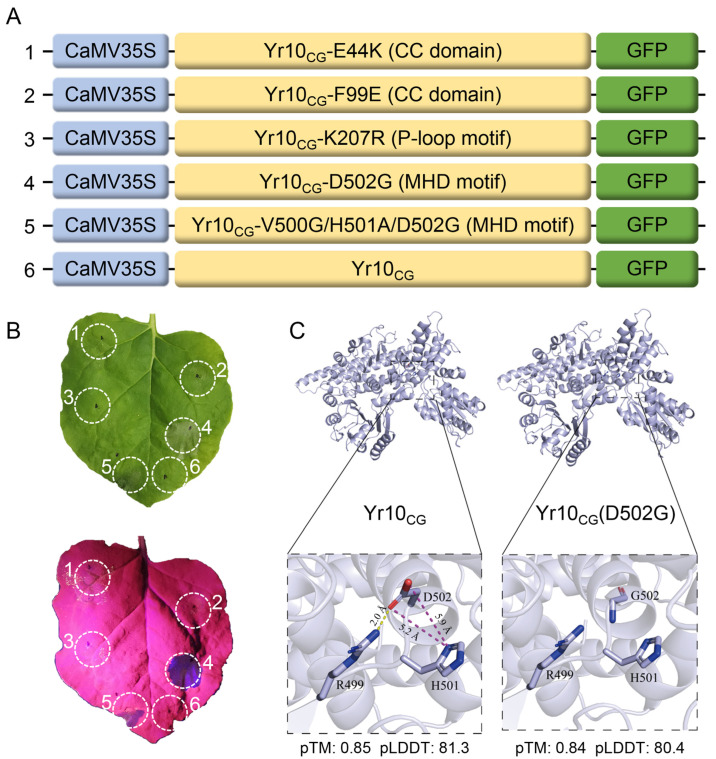
MHD motif mutations in Yr10_CG_ trigger autoactive cell death in *Nicotiana benthamiana*. (**A**) Schematic diagram of expression vectors for Yr10_CG_ and mutated sites in Yr10_CG_, each fused to GFP. (**B**) *Agrobacterium* (GV3101) containing the Gateway constructs from (**A**) was infiltrated into 6-week-old *Nicotiana benthamiana* leaves. Hypersensitive responses were detected at 24 hpi. The cell death was visualized under excitation at 440–460 nm using the LUYOR-3415RG Hand-Held Lamp with a 500 nm filter. (**C**) The tertiary structural model of Yr10_CG_ and Yr10_CG_-D502G predicted by AlphaFold 2 highlights changes in molecular interactions in the MHD motif region. The predicted distance between guanidino group protons in the side chain of the Arginine (R499) and the carboxyl oxygens of the Aspartate (D502) is 2 Å. The predicted distances between the carboxyl oxygens of the Aspartate (D502) and the hydrogen atoms of the imidazole ring of the Histidine (H501) are 5.2 Å and 5.9 Å. The predicted template modeling (pTM) and predicted local distance difference test (pLDDT) confidence scores are included to indicate the quality of the AlphaFold2 predicted structures.

The MHD motif of Yr10_CG_ is located from residues 500 to 502 with a sequence of VHD. We generated two mutants of this MHD motif: one with a single mutation changing D502 to G and another substituting VHD at positions 500–502 with GAG (Figure 5A). Our study found that the transient expression of both MHD motif mutants in *Nicotiana benthamiana* leaves led to cell death (Figure 5B), suggesting that mutations in the MHD motif may cause a conformational change in Yr10_CG_, leading to self-activation and triggering immune responses.

Using AlphaFold 2, we predicted the structures of Yr10_CG_ and Yr10_CG_-D502G and compared the intramolecular interactions of their MHD motif region (Figure 5C). In Yr10_CG_, the side chain of D502 engages in a hydrogen bond with R499’s side chain and electrostatic interaction with H501’s side chain. However, the single-point mutation of D502 to glycine (G) in Yr10_CG_(D502G) disrupts all these interactions, potentially destabilizing the protein and increasing local flexibility.

Additionally, single mutation constructs were also introduced in Yr10_CG_ at E44K and F99E (both within the CC domain) and K207R (within the P-loop motif) and fused with GFP (Figure 5A). When transiently expressed in *Nicotiana benthamiana* leaves, these mutants displayed phenotypes similar to Yr10_CG_ and did not trigger cell death like the MHD motif mutants (Figure 5B).

### 2.4. CC Domain of Yr10CG Does Not Involve Recognizing the Effector, but for Activating the Downstream Immunity Mechanism

**Figure 6:**
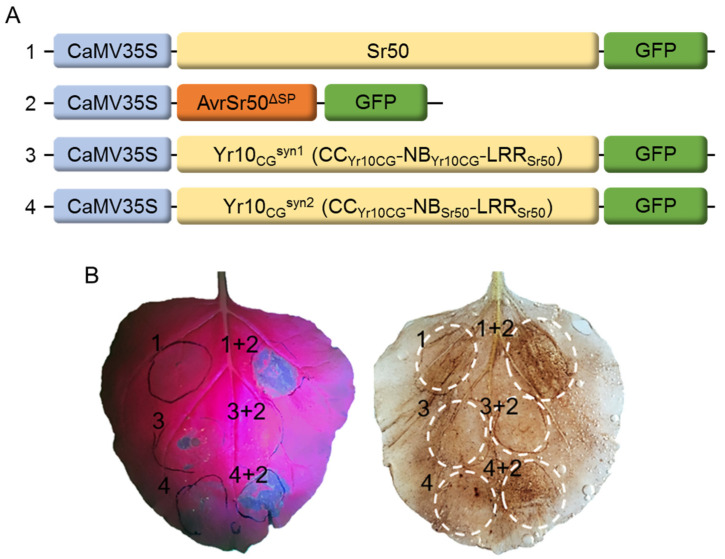
Transient expression of Sr50 chimeric Yr10_CG_ constructs co-expressed with AvrSr50^ΔSP^ in *Nicotiana benthamiana*. (**A**) Schematic diagram of expression vectors for Sr50, AvrSr50^ΔSP^, and Yr10_CG_^syn1^ (comprising the CC and NB-ARC domains from Yr10_CG_ and the LRR domain from Sr50) and Yr10_CG_^syn2^ (comprising the CC domain from Yr10_CG_ and the NB-ARC and LRR domains from Sr50). (**B**) Infiltration of 5-week-old *Nicotiana benthamiana* leaves with *Agrobacterium* (GV3101) containing the Gateway constructs from (**A**). Hypersensitive responses were detected at 24 hpi. Cell death was visualized under excitation at 440–460 nm using the LUYOR-3415RG Hand-Held Lamp with a 500 nm filter and was confirmed by DAB staining.

We wanted to explore whether Yr10_CG_ could be modified to recognize a race-specific pathogen effector. Studies indicate that the LRR domain is pivotal in recognizing pathogen effectors. Consequently, we substituted the LRR domain of Yr10_CG_ with that of Sr50 to engineer a chimeric NLR named Yr10_CG_^syn1^ (Figure 6A) and tested its ability to recognize AvrSr50. When Yr10_CG_^syn1^ and AvrSr50 were transiently co-expressed in *Nicotiana benthamiana* leaves, no cell death was observed, contrary to the expected response with the Sr50/AvrSr50 pair (Figure 6B). This suggests that the recognition of AvrSr50 by Sr50 likely requires cooperation between both the LRR and NB-ARC domains. To further investigate, we created another chimeric NLR, Yr10_CG_^syn2^ (Figure 6A), by replacing both the NB-ARC and LRR domains of Yr10_CG_ with those from Sr50. When Yr10_CG_^syn2^ and AvrSr50 were transiently co-expressed in *Nicotiana benthamiana* leaves, we observed cell death, similarly to the response expected and observed with Sr50 and AvrSr50 (Figure 6B). This finding supports the requirement of the NB-ARC and LRR domains to function together for the recognition of AvrSr50. The results also suggest that the Yr10_CG_ CC domain can substitute for the native CC domain of Sr50, undergoing a similar conformational change to execute immune signaling after activation by AvrSr50.

**Figure 7:**
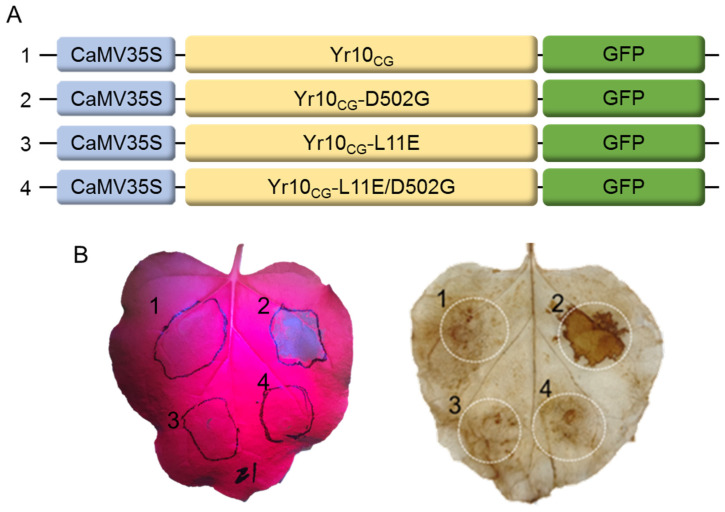
The L-to-E mutation at position 11 in Yr10_CG_ suppresses autoactive cell death caused by MHD motif mutations in *Nicotiana benthamiana*. (**A**) Schematic diagram of expression vectors for Yr10_CG_ and mutated sites in Yr10_CG_, each fused to GFP. (**B**) Infiltration of 5-week-old *Nicotiana benthamiana* leaves with *Agrobacterium* (GV3101) containing the Gateway constructs from (**A**). Hypersensitive responses were detected at 24 hpi. Cell death was visualized under excitation at 440–460 nm using the LUYOR-3415RG Hand-Held Lamp with a 500 nm filter and was confirmed by DAB staining.

Recent structural studies on ZAR1 and Sr35 have shown that the CC domain plays a critical role in membrane localization and the formation of a pore [26,27,45], which subsequently triggers calcium influx [32]. We demonstrated that the CC domain of Yr10_CG_, similarly to that of Sr50, can mediate immune signaling when the NLR recognizes an effector (Figure 6B). We hypothesized that, like ZAR1 and Sr35, Yr10_CG_ might form oligomeric structures needed for a resistosome at the cell membrane upon activation, possibly generating ion channels that trigger downstream immune signaling. This led us to consider introducing a mutation in the CC domain to interfere with the immune function via inept cell death. To test this, we utilized the confirmed self-activating Yr10_CG_-D502G mutant. We introduced an additional mutation at residue 11, changing the hydrophobic leucine (L) to the hydrophilic glutamic acid (E), creating the double mutant Yr10_CG_-L11E/D502G (Figure 7A), which may hinder the interaction of CC domains with lipid components of the plasma membrane. When Yr10_CG_-L11E/D502G was transiently expressed in *Nicotiana benthamiana* leaves, the cell death activity observed with Yr10_CG_-D502G alone (Figure 7B) was eliminated. This indicates that the L11E mutation suppresses the self-activation of Yr10_CG_-D502G, likely due to impaired membrane localization of the CC domain. We attempted to predict the level and the nature of the possible structural changes introduced by the mutations using AlphaFold3.

**Figure 8:**
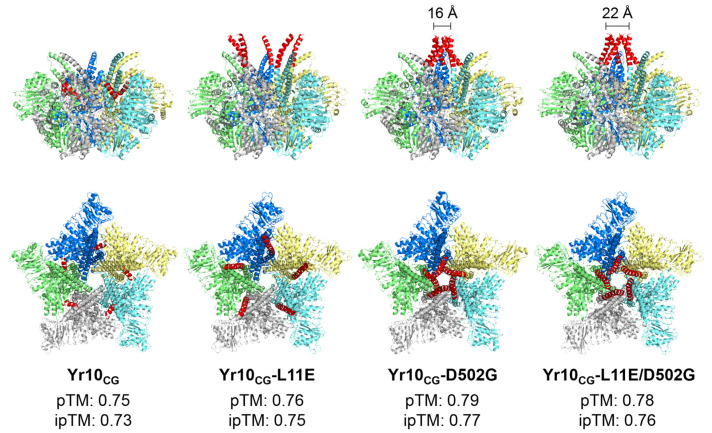
Pentameric structures of Yr10_CG_ and its mutants predicted by AlphaFold 3. Each monomer is shown in a distinct color for clarity, while the α1 helices of CC domains are highlighted in red. Both the side view and top view of each pentameric structure are presented. The measured distances above the predicted pentamer structures of Yr10_CG_-D502G and Yr10_CG_-L11E/D502G indicate the diameter of the pore formed by the five C^α^ atoms at “position 1”, corresponding to the first amino acid in the N-terminus of each monomer. The predicted template modeling (pTM) and interface pTM (ipTM) confidence scores are included to indicate the quality of the AlphaFold3 predicted structures.

Activated CNL resistosomes can insert themselves into cell membranes through a funnel-like structure formed by their N-terminal α1 helices, ultimately triggering cell death [54]. Recent studies using AlphaFold 3, a newly released tool for predicting the structures of activated CNL oligomers, have demonstrated the reliable modeling of CNLs and their α1 helices [55]. Using AlphaFold 3, we predicted the pentameric structures of Yr10_CG_, Yr10_CG_-L11E, Yr10_CG_-D502G, and Yr10_CG_-L11E/D502G (Figure 8). These predictions correlate well with our experimental results. In the predicted structure of Yr10_CG_, the α1 helices are embedded within the pentamer’s interior, preventing membrane insertion. In Yr10_CG_-L11E, while the α1 helices do extend outward, they fail to cluster tightly, which likely hinders membrane insertion and immune activation. In contrast, the α1 helices of Yr10_CG_-D502G successfully form a funnel-like structure similar to that seen in ZAR1, consistent with our experimental results that show cell death induction in this variant. For Yr10_CG_-L11E/D502G, the α1 helices are clustered, but experimental data indicate that the L11E mutation suppresses the autoactivation caused by D502G. The predicted structure suggests that the pore diameter formed by the C^α^ atoms at the first amino acid in the N-terminus of each monomer in Yr10_CG_-L11E/D502G is wider than that of Yr10_CG_-D502G. This difference in pore size, coupled with the impaired membrane localization caused by the L11E mutation, may contribute to reduced downstream immune signaling despite the maintenance of the pore.

## 3. Discussion

Our findings shed light on the intricate regulatory mechanisms governing the wheat CC-type NLR Yr10_CG_ and underscore the potential of NLRs for genetic engineering in crop protection. The mechanism of resistance of CC-NLRs was first discovered in the ZAR1 study and presented via invaluable cryo-electron microscopy (cryo-EM)-based structure determination [26,27]. The robust autoinhibition inferred in Yr10_CG_ aligns with findings in other CC-NLRs and suggests that a high level of structural integrity is necessary to maintain the receptor in its inactive state until activation is triggered. In our study, AlphaFold3 predicted a presumed pentameric resistosome. We must emphasize that these structures are only predictions until determination via X-ray or cryo-EM structures confirms them. Currently, we know that this gene is not the true *Yr10*; in other words, it does not recognize AvrYr10. However, it may be cognate to another Avr, or it may be a pseudogene. Thus, the structure should be experimentally confirmed by cryo-EM after the gene is identified through pathogenicity experiments or synthetically engineered to confer resistance to specific pathogen races. However, the predicted disruption to key interactions within the MHD motif by the D502G mutation, leading to local flexibility and activation, offers a plausible explanation for Yr10_CG_ self-activation. The D502G mutation in the MHD motif triggers activation by destabilizing key interactions that maintain the inactive state, similarly to activation mechanisms observed in NLRs like ZAR1 and Sr35 [26,27,45,46]. This mutation-induced flexibility may disrupt autoinhibition and promote ATP binding [56], which shifts Yr10_CG_ to an active state, thereby initiating an immune response.

Our chimeric NLR experiments reveal that the NB-ARC and LRR domains collectively facilitate effector recognition, while the CC domain primarily contributes to downstream signaling. This division of labor between domains is consistent with models in which LRR domains are essential in specific effector binding, while the CC domain plays a signaling role post recognition. In the study by Tamborski et al., the engineered Sr33 gained the ability to recognize AvrSr50 through just 12 amino acid substitutions in the LRR domain [57]. This is likely due to the high sequence similarity between Sr33 and Sr50. In contrast, our engineered Yr10_CG_ achieved the recognition of AvrSr50 by replacing both the NB-ARC and LRR domains. The requirement for membrane localization for Yr10_CG_ activation aligns with recent structural findings, suggesting that CC domains in NLRs may form higher-order complexes at the membrane, which function as ion channels or signaling platforms upon activation [32,45]. This mechanism highlights the complex regulation of NLR signaling, where spatial organization is as crucial as structural integrity in effective immune response initiation. Although *Yr10_CG_* is not a resistance gene against Yr10 virulence, its protein sequence is highly similar to wheat stem rust resistance proteins Sr33, Sr35, and Sr50 and barley mildew resistance protein MLA10. It would be interesting to test whether Yr10_CG_ presents any resistance phenotype against different races of stripe rusts with known pathogenicity in transgenic wheat with *Yr10_CG_*, along with races of stem rust and powdery mildew, to explore whether this candidate R-gene plays a role in resistance to wheat stem rust and powdery mildew. Such a study may help in determining whether it functions as a different R-gene.

Our transient expression experiments in *Nicotiana benthamiana* provide initial evidence for the activation and signaling mechanisms of Yr10_CG_ mutants. Nevertheless, it would be preferable to demonstrate the results for the mutants in the physiological and genetic context of wheat. Future studies could focus on generating stable transgenic wheat lines carrying these mutants to evaluate their role in plant resistance under controlled and field conditions. The development of the stable transformation of these mutants will help confirm whether the mechanisms observed in this study are consistent with the native biological function of these mutants in the target crop. In addition to molecular modeling and computational analyses, future studies could explore experimental approaches such as directed evolution [58] or phage display [59] to engineer novel R-genes with the ability to recognize a broader spectrum of *Pst* races. Furthermore, already-characterized integrated domains of NLRs, such as BED or WRKY domains, could be incorporated into Yr10_CG_ through domain swaps or fusions to create engineered receptors with enhanced pathogen recognition capabilities [60,61].

Altogether, this study deepens our understanding of Yr10_CG_ and the broader class of CC-type NLRs, offering a basis for targeted modifications to enhance immune responses in wheat. The insights gained here suggest that the rational design and manipulation of NLR domains may allow for the more precise engineering of immune receptors with tailored activation profiles, a promising strategy for durable pathogen resistance in crops. Further research may explore additional structural adjustments or combinatorial mutations in Yr10_CG_ to achieve novel effector recognition capabilities, broadening the applications of NLR-based immunity in crop breeding.

## 4. Materials and Methods

### 4.1. Plasmid Construction

The genes *Yr10_CG_* (AF149112), *Sr50* (QNU41030.1), and *AvrSr50* (as identified by Chen et al., 2017 [62]) were cloned by GenScript Biotech Corporation (Nanjing, China) into the pUC57_Mini vector to serve as templates for subsequent cloning.

Using Q5 high-fidelity DNA polymerase (NEB#E0555S, Ipswich, MA, USA), the fragments of Yr10_CG_, the CC domain of Yr10_CG_ (135 amino acids, designated as CC_Yr10CG_), Sr50, and AvrSr50 without the signal peptide (AvrSr50^ΔSP^) were amplified through PCR. These PCR products were then inserted individually into the pENTR TOPO vector (Invitrogen#45-0218, Carlsbad, CA, USA) via the TOPO Cloning reaction according to the manufacturer’s protocol. Additionally, several pENTR TOPO constructs containing variants of Yr10_CG_ were generated by site-directed mutagenesis via a PCR-driven overlap technique [63], including the following constructs: Yr10_CG_-L11E, Yr10_CG_-E44K, Yr10_CG_-F99E, Yr10_CG_-K207R, Yr10_CG_-D502G, Yr10_CG_-L11E/D502G, and Yr10_CG_-V500G/H501A/D502G. The primers used in these reactions are listed in Table 1. In addition, GenScript synthesized two chimeric constructs, Yr10_CG_^syn1^ (comprising the CC and NB-ARC domains from Yr10_CG_ and the LRR domain from Sr50) and Yr10_CG_^syn2^ (comprising the CC domain from Yr10_CG_ and the NB-ARC and LRR domains from Sr50), which were also cloned into the pENTR TOPO vector.

These pENTR TOPO constructs were subsequently transferred into the Gateway compatible binary vector pGWB505 (Addgene#74847) using the LR Clonase II enzyme mix kit (Invitrogen#11791-020, Carlsbad, CA, USA) according to the manufacturer’s protocol, resulting in C-terminal GFP fusion with the expressed genes. Similarly, Yr10_CG_ and CC_Yr10CG_ were also cloned into pGWB514 (Addgene#74856) through the same LR reaction, resulting in C-terminal fusion with a 3×HA tag for these constructs.

### 4.2. Agrobacteria-Mediated Transient Expression in Nicotiana benthamiana

*Nicotiana benthamiana* plants were cultivated at 23 °C under a 16 h light/8 h dark cycle in a growth chamber. GFP or 3×HA fusion constructs were introduced into competent cells of *Agrobacterium tumefaciens* strain GV3101 (WEIDI#AC1001, Shanghai, China) by heat shock transformation [64]. The transformed *Agrobacteria* GV3101 were then grown for 2 d at 28 °C on a Luria–Bertani (LB) plate with 10 μg/mL rifampicin (Sangon#A600812, Shanghai, China) and 100 μg/mL spectinomycin hydrochloride (Sangon#A600901, Shanghai, China). Positive colonies were inoculated in 5 mL of LB liquid medium with the same antibiotics and cultured at 28 °C in the dark for 36 h with shaking at 200 rpm. The bacterial cultures were then centrifuged at 5000× *g* to collect cells and resuspended in Agroinfiltration buffer (10 mM MES (Solarbio#M8010, Beijing, China) pH 5.7, 10 mM MgCl_2_ (Sangon#A610328, Shanghai, China), 0.1 mM acetosyringone (Sigma-Aldrich#D134406, Saint Louis, MO, USA)). The final optical density (OD_600nm_) for all suspensions was adjusted to 0.7, and the suspensions were incubated at room temperature in the dark for 3 h prior to infiltration. Leaves from 5- to 6-week-old *N. benthamiana* plants were selected for infiltration, which was performed by injecting the bacterial suspensions into the leaves using a 1 mL syringe without a needle. For each construct, at least 6 individual leaves from different plants were infiltrated.

### 4.3. Hypersensitive Response Assay

*Nicotiana benthamiana* leaves at 24 h post infiltration (hpi) was used for hypersensitive response (HR) assay. The HR is identified by visible necrosis and the presence of autofluorescence [65]. To capture the fluorescence associated with this response, the leaves were illuminated with a 440–460 nm excitation light from the LUYOR-3415RG Hand-Held Lamp (Luyor, Shanghai, China) and photographed through a 500 nm filter.

The parts that produce hydrogen peroxide in the leaves can be stained dark brown by DAB (3′,3-diaminobenzidine) [66]. *Nicotiana benthamiana* leaves at 24 hpi were collected and soaked overnight in a DAB staining solution (10 mM MES buffer at pH 3.8 (Solarbio#M8010, Beijing, China) and 2 mM DAB (Macklin#D807021, Shanghai, China)). Following staining, the leaves were decolorized by soaking in a mixture of glycerol, acetic acid, and ethanol (in a 1:1:3 volume ratio) at 60 °C until the green pigment had completely faded.

### 4.4. Bioinformatics Analysis

Protein sequences of cloned NLR genes from *Triticeae* were collected and subjected to multiple sequence alignment using Clustal Omega (https://www.ebi.ac.uk/jdispatcher/msa/clustalo, accessed on 7 November 2024) [67]. A phylogenetic tree was generated from these sequences using iTOL (https://itol.embl.de, accessed on 7 November 2024) [68]. The multiple sequence alignment results for Yr10_CG_, Sr35, MLA10, Sr50, and Sr33 were further processed to generate an alignment visualization in ESPript 3.0 (https://espript.ibcp.fr/ESPript/cgi-bin/ESPript.cgi, accessed on 8 November 2024) [69]. Key motifs in Yr10_CG_, predicted using NLRexpress (https://nlrexpress.biochim.ro/, accessed on 8 November 2024) [70], were annotated on the alignment. For structural analysis, protein structure predictions of Yr10_CG_ and the Yr10_CG_-D502G were conducted using AlphaFold2 (https://colab.research.google.com/github/sokrypton/ColabFold/blob/main/AlphaFold2.ipynb, accessed on 15 June 2023) [71]. Oligomerization structure predictions for Yr10_CG_ and its variants were performed using AlphaFold3 (https://alphafoldserver.com/, accessed on 13 May 2024) [72]. The predicted structures were visualized and edited in PyMOL 2.0 (Delano Scientific: San Carlos, CA, USA) [73].

## 5. Conclusions

Yr10_CG_ exhibits a robust autoinhibitory mechanism, neither when assuming native confirmation within the cell nor its CC domain alone triggers cell death in *Nicotiana ben-thamiana*. However, the D502G mutation in the conserved MHD motif disrupts this inhibition and induces self-activation. Our experiments with chimeric NLRs demonstrate that the NB-ARC and LRR domains of Sr50 are essential in effector recognition, while the CC domain of Yr10_CG_ functions in downstream immune signaling. Additionally, we found that membrane insertion is critical in Yr10_CG_ activation, and that the L11E mutation may disrupt membrane association and suppress its immune response.

## Figures and Tables

**Table 1:** Primers used for vector constructions.

	Primers	Sequence (5′-3′)
PCR product for TOPO cloning reaction	CACC-Yr10_CG_/CC_Yr10CG_ F	caccatggaggtcgtgaccggg
	Yr10_CG_ R	gcgtggagttaccttcaccgt
	CC_Yr10CG_ R	gtcactgacctccttgatgcggc
	CACC-Sr50 F	caccatgaatattgtcacgggggccatg
	Sr50 R	gttctcctcacacaaatcatcatcacgag
	CACC-AvrSr50^ΔSP^ F	caccatggctaggagccttgtcaaaattg
	AvrSr50^ΔSP^ R	cctgtgttggcgccttgc
Site-directed mutagenesis on pENTR TOPO-Yr10_CG_	Yr10_CG_-L11E F	atgagcacggaactgcccttg
	Yr10_CG_-L11E R	caagggcagttccgtgctcat
	Yr10_CG_-E44K F	gagagcatgaaggctgccctcatcaagatc
	Yr10_CG_-E44K R	agggcagccttcatgctctccagctctg
	Yr10_CG_-F99E F	gccacacagcgaaatgggtttcatccacaa
	Yr10_CG_-F99E R	tgaaacccatttcgctgtgtggcttcttttgt
	Yr10_CG_-K207R F	gcttagggagaacaactcttgctaacgtggta
	Yr10_CG_-K207R R	caagagttgttctccctaagcctccaaagcc
	Yr10_CG_-D502G F	gtacacggaatggtgcttgaccttatcac
	Yr10_CG_-D502G R	gcaccattccgtgtacacggacagagctc
	Yr10_CG_-V500G/H501A/D502G F	tctgtccgtggagctggaatggtgcttgacct
	Yr10_CG_-V500G/H501A/D502G R	agcaccattccagctccacggacagagctcg

## Data Availability

The analysis results of all data are presented in the text.
